# Molecular and genetic alterations associated with therapy resistance and relapse of acute myeloid leukemia

**DOI:** 10.1186/s13045-017-0416-0

**Published:** 2017-02-20

**Authors:** Hubert Hackl, Ksenia Astanina, Rotraud Wieser

**Affiliations:** 1 Division of Bioinformatics, Biocenter, Medical University of Innsbruck, Innrain 80, 6020 Innsbruck, Austria; 2 Department of Medicine I and Comprehensive Cancer Center, Medical University of Vienna, Währinger Gürtel 18-20, 1090 Wien, Austria

**Keywords:** Acute myeloid leukemia, Relapse, Therapy resistance, Clonal evolution, Cytogenetics, Copy number variation, Single nucleotide variants, DNA methylation, Gene expression profiling

## Abstract

**Background:**

The majority of individuals with acute myeloid leukemia (AML) respond to initial chemotherapy and achieve a complete remission, yet only a minority experience long-term survival because a large proportion of patients eventually relapse with therapy-resistant disease. Relapse therefore represents a central problem in the treatment of AML. Despite this, and in contrast to the extensive knowledge about the molecular events underlying the process of leukemogenesis, information about the mechanisms leading to therapy resistance and relapse is still limited.

**Purpose and content of review:**

Recently, a number of studies have aimed to fill this gap and provided valuable information about the clonal composition and evolution of leukemic cell populations during the course of disease, and about genetic, epigenetic, and gene expression changes associated with relapse. In this review, these studies are summarized and discussed, and the data reported in them are compiled in order to provide a resource for the identification of molecular aberrations recurrently acquired at, and thus potentially contributing to, disease recurrence and the associated therapy resistance. This survey indeed uncovered genetic aberrations with known associations with therapy resistance that were newly gained at relapse in a subset of patients. Furthermore, the expression of a number of protein coding and microRNA genes was reported to change between diagnosis and relapse in a statistically significant manner.

**Conclusions:**

Together, these findings foster the expectation that future studies on larger and more homogeneous patient cohorts will uncover pathways that are robustly associated with relapse, thus representing potential targets for rationally designed therapies that may improve the treatment of patients with relapsed AML, or even facilitate the prevention of relapse in the first place.

**Electronic supplementary material:**

The online version of this article (doi:10.1186/s13045-017-0416-0) contains supplementary material, which is available to authorized users.

## Background

Acute myeloid leukemia (AML) is a malignant disease of hematopoietic stem and progenitor cells (HSPCs) with a median age of onset of around 67 years and an annual incidence of 3–8/100.000 [1–4]. It is characterized by the accumulation of immature blasts at the expense of normal, functional myeloid cells in the bone marrow and peripheral blood of affected patients. Standard induction chemotherapy, based on cytosine arabinoside and an anthracycline like daunorubicin or idarubicin, leads to complete remissions (CRs) in 40 to >90% of cases, depending on patient age and the presence or absence of specific somatically acquired genetic alterations [1–6]. Together with post-remission therapy (additional chemotherapy and/or hematopoietic stem cell transplantation), 5-year survival rates of <5–20 and >40% are achieved for patients older and younger than 60 years, respectively [1–4, 7]. Patients with acute promyelocytic leukemia (APL), which is driven by fusion proteins involving the retinoic acid receptor alpha (RARA), fare substantially better than other patients with AML: in response to targeted therapy based on *all-trans* retinoic acid, combined with cytosine arabinoside or arsenic trioxide, they achieve CR and long-term remission rates of >90 and >80%, respectively [8, 9].

The discrepancy between the favorable primary response rates and the substantially lower long-term survival rates in AML is due to the fact that a high proportion of patients who achieve CR eventually relapse [2, 5, 6]. Even though second and even third remissions may be achieved, these are of progressively shorter duration, and cure is rarely accomplished. Relapse, and the associated resistance to currently available therapies, therefore represents one of the central problems in the treatment of AML [2, 6, 7, 10].

Similar to normal hematopoiesis, leukemic hematopoiesis is organized in a hierarchical manner. The bulk of the leukemic cell mass is derived from mostly quiescent leukemic stem cells (LSCs), which can divide either symmetrically to produce two stem cells, or asymmetrically to give rise to one stem cell and one more differentiated progenitor cell [11, 12]. The transforming events giving rise to an LSC may take place either in a hematopoietic stem cell (HSC), or in a progenitor cell that consequently regains stem cell characteristics [11, 12]. Like their healthy counterparts, LSCs reside in the bone marrow niche, and interactions with stromal cells in this niche promote LSC dormancy and protection from chemotherapy [11, 12]. The frequency of LSCs is measured mainly through transplantation experiments; estimates range from 1 in 500 to 1 in 10^7^ cells, depending both on experimental variables and on leukemia-intrinsic factors. In agreement with LSCs representing a bastion of therapy resistance and a potential source of relapse, high LSC frequencies, as well as the presence of a stem cell expression signature, correlate with inferior outcome in AML [11–13]. On the other hand, since up to 40% of patients with AML are cured by conventional therapies, LSCs are not resistant to these in all cases. A variety of different and only partially explored factors contribute to the therapy refractoriness of LSCs, which may be considered their clinically most relevant property [11].

Like other malignant diseases, AML is the result of somatically acquired genetic lesions, e.g., numerical and structural chromosome aberrations, copy number alterations (CNAs), uniparental isodisomies (UPDs), small insertions or deletions (indels), and single nucleotide variants (SNVs)[5, 14–19], which accumulate in LSCs and consequently are present also in their progeny. In addition, epigenetic and transcriptional changes contribute to leukemogenesis [5, 15–17, 20–25]. Aberrations present in the malignant cells of different patients (i.e., recurrent alterations) are assumed and, in many cases, have been shown to act as drivers of leukemogenesis. They serve as useful prognostic markers [14–19, 26] and additionally may represent suitable targets for rationally designed therapies [5, 8, 9, 27–29].

Recently, next generation sequencing-based investigations have yielded important novel insights into the molecular pathogenesis of AML. They have uncovered previously unknown recurrent aberrations in this disease entity [30, 31] and revealed that AML genomes on average contained several hundred mutations in non-repetitive regions but only low two-digit numbers of mutations with predicted translational consequences, which is substantially fewer than in most solid tumor genomes [17, 32–34]. An even smaller number of mutations per patient affected suspected leukemogenic driver genes. These appeared to accumulate in a specific order, in that mutations in genes coding for epigenetic regulators and chromatin remodeling factors tended to occur early, while mutations in genes coding for transcription factors and signaling molecules typically arose late in the process of leukemogenesis [19, 35–38]. Remarkably, early mutations were also found in phenotypically and functionally normal HSCs in a substantial proportion of AML patients, and often persisted in remission [35–39]. Furthermore, a subset of healthy individuals exhibited low levels of clonal hematopoiesis that could, but did not necessarily, involve early leukemogenic driver mutations [40–42]. The frequency of this phenomenon, termed “clonal hematopoiesis of indeterminate potential” (CHIP), increased strongly with age, and the affected persons carried a substantially increased, albeit in absolute terms still low, risk to develop hematological malignancies [40–43]. Overall, a picture emerges in which HSCs accumulate mutations during the lifetime of an individual. Some of these lesions lead to the formation of preleukemic stem cells, which have a proliferation and/or survival advantage but are still able to give rise to functional, differentiated progeny. Additional mutations, often in genes coding for signaling proteins or transcription factors, are required to promote the transformation to LSCs and, consequently, overt AML [44, 45]. This mutational history is reflected in the clonal composition of AML samples. Based on the distributions of variant allele frequencies (VAFs) of individual mutations, diagnostic AML samples were found to harbor 1–4 cellular clones whose size exceeded the detection threshold of the employed methods. In oligoclonal cases, a founding clone contained the age-related and pathogenetically probably largely irrelevant majority of the sequence variants, as well as the early leukemic driver mutations, at VAFs indicating their presence in almost all cells of the sample. One to three subclones harbored additional mutation clusters, including late driver mutations, at lower VAFs [17, 32–34]. Further minor subpopulations were often detectable upon application of more sensitive methods [36, 46, 47].

**Fig. 1:**
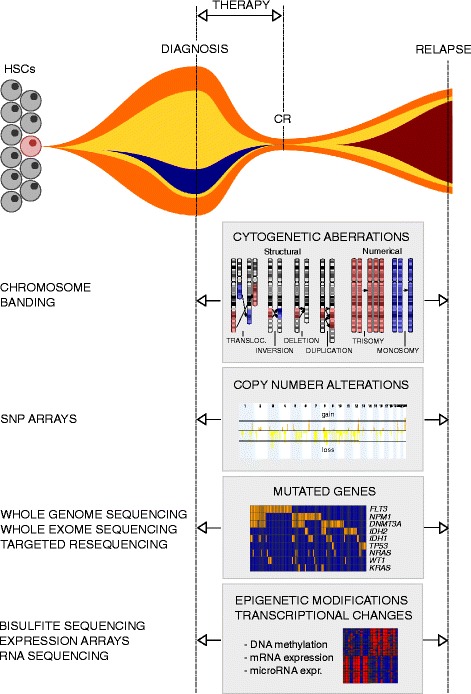
Genetic and molecular events investigated for possible changes between diagnosis and relapse of AML. A diagram representing clonal evolution in a hypothetical patient with AML is shown in the *top panel*. The other panels represent genetic and molecular alterations between diagnosis and relapse of AML that are discussed in this article; methods used to investigate these aberrations are indicated to the *left* of the respective panels. *HSCs* hematopoietic stem cells, *CR* complete remission, *transloc* translocation, *SNP* single nucleotide polymorphism

The majority of genetic and molecular studies on AML have focussed on the characterization of alterations present at the time of diagnosis, yet, as outlined above, a large proportion of AML patients with primarily responsive disease ultimately die due to relapse with refractory leukemia. The survival of stem cells, whose regrowth leads to disease recurrence, is assumed to be due in part to protective effects of the microenvironment [48–50] and in part to cell autonomous mechanisms elicited by molecular alterations in the stem cells themselves, as has been impressively demonstrated in the case of acute lymphoblastic leukemia [51, 52]. Such molecular changes may already have been present in a (sometimes very small) subset of stem cells at presentation, or may have emerged during, and even as a consequence of the mutagenic effects of, cytostatic therapy [34, 39]. For specific lesions to qualify as candidate drivers of relapse, they should (1) be recurrently gained at this disease stage (for the purpose of this review, the definition of “gain” or “acquisition” at relapse includes a strong increase in abundance), (2) not be lost at relapse in other patients (albeit cells carrying a molecular alteration capable of conferring therapy resistance might be outcompeted by cells with an even stronger selective advantage in a small number of cases), and (3) either not be observed at diagnosis, or be associated with poor response to therapy if present at this stage. A still limited but rapidly growing number of investigations have assessed genetic, epigenetic, and gene expression differences in AML cells from the times of diagnosis and relapse (Fig. 1). In order to further explore mechanisms that may lead to therapy resistance and relapse in AML, these studies are summarized in this review, and data from them are compiled into comprehensive tables (Table 1, Additional file 1: Table S1, Additional file 2: Table S2 and Additional file 3: Table S3).

**Table 1:** Gains and losses of mutations in known leukemia driver genes at relapse of AML

	Total number of patients	Age group	Genetics at diagnosis	Number of patients with gain of mutation	Number of patients with loss of mutation	Reference
***FLT3-ITD***						
**Total**	**492**			**38**	**25**	
	28	A		1	1	[65]
	28	A		6	1	[77]
	34	A		2	3	[76]
	108	A		8	1	[81]
	31	A		1	2	[85]
	53	A	*NPM1* ^m^	9	3	[69]
	80	A, P		5	4	[79]
	44	A, P		2	5	[80]
	23	P		2	1	[84]
	63	P		2	4	[83]
***FLT3-TKD***						
**Total**	**385**			**10**	**24**	
	34	A		1	3	[76]
	120	A		6	8	[82]
	31	A		0	1	[85]
	53	A	*NPM1* ^m^	0	10	[69]
	53	A, P		0	1	[79]
	44	A, P		2	0	[80]
	50	P		1	1	[83]
***NPM1***						
**Total**	**299**			**0**	**9**	
	28	A		0	0	[65]
	34	A		0	3	[76]
	53	A	*NPM1* ^m^	n.a.	5	[69]
	70	A, P	*NPM1* ^m^	n.a.	0	[124]
	46	P		0	0	[125]
	68	P		0	1	[83]
***DNMT3A***						
**Total**	**231**			**1**	**2**	
	28	A		0	0	[65]
	34	A		0	0	[76]
	116	A		0	1	[87]
	53	A	*NPM1* ^m^	1	1	[69]
***CEBPA***						
**Total**	**241**			**2**	**5**	
	28	A		1	1	[65]
	34	A		0	2	[76]
	149	A, P		0	2	[86]
	30	P		1	0	[83]
***IDH2***						
**Total**	**236**			**0**	**1**	
	28	A		0	0	[65]
	34	A		0	0	[76]
	121	A		0	0	[126]
	53	A	*NPM1* ^m^	0	1	[69]
***IDH1***						
**Total**	**115**			**4**	**2**	
	28	A		0	0	[65]
	34	A		0	0	[76]
	53	A	*NPM1* ^m^	4	2	[69]
***NRAS***						
**Total**	**106**			**8**	**12**	
	19	A		2	3	[77]
	34	A		1	0	[76]
	53	A	*NPM1* ^m^	5	9	[69]
***KRAS***						
**Total**	**62**			**1**	**1**	
	28	A		0	1	[65]
	34	A		1	0	[76]
***RAS***						
**Total**	**75**			**6**	**8**	
	23	P		2	1	[84]
	52	P		4	7	[83]
***TP53***						
**Total**	**104**			**3**	**1**	
	28	A		0	1	[77]
	23	A		2	0	[78]
	53	A	*NPM1* ^m^	1	0	[69]
***WT1***						
**Total**	**104**			**14**	**0**	
	23	P		3	0	[84]
	42	P		5	0	[83]
	39	P		6	0	[127]
***ASXL1***						
**Total**	**81**			**2**	**0**	
	28	A		0	0	[65]
	53	A	*NPM1* ^m^	2	0	[69]
***KIT***						
**Total**	**35**			**0**	**0**	
	27	P		0	0	[83]
	8	P	CBF	0	0	[128]
***TET2***						
**Total**	**62**			**0**	**0**	
	28	A		0	0	[65]
	34	A		0	0	[76]
***MLL-PTD***						
	34	A		0	0	[76]
***PTPN11***						
	23	P		0	1	[84]
***RUNX1***						
	28	A		1	1	[65]

The total number of investigated patients, patient age group, genetics at diagnosis in studies based on selected samples, the number of patients with gain or loss of mutation in the respective gene, and the corresponding references are listed. This table summarizes mutations determined by small scale targeted sequencing approaches. Gains and losses of mutations in these genes were also found through next generation sequencing-based methods, as summarized in Additional file 3: Table S3A and B

*A* adult, *P* pediatric, *n.a.* not applicable, *NPM1* ^*m*^ AML with *NPM1* mutations, *CBF* AML with core-binding factor rearrangements

## Cytogenetic changes between diagnosis and relapse of AML

Cytogenetics yielded the first insights into leukemia genetics, and cytogenetic analyses were the first to compare leukemic samples from the times of presentation and recurrence. During progression to relapse, karyotypes developed following five major patterns: no change (stability), acquisition of additional alterations (progression or evolution), loss of alterations (regression or devolution), progression combined with regression, and the emergence at recurrence of karyotypes that were unrelated to those found at presentation. Studies including 45–168 patients with AML observed a stable karyotype in 39-62% of them [53–56]. Among the different types of karyotypic instability, evolution was present in 25–46% of all patients, and devolution, evolution + devolution, and unrelated karyotypes at relapse were observed in 13–22, 5–12, and 2–8% of cases with an abnormal karyotype at diagnosis, respectively [53, 54, 56]. In one patient cohort, normal karyotypes appeared to be more stable than abnormal karyotypes [54], while in another, normal karyotypes and abnormal karyotypes exhibited similar frequencies of evolution, and only patients with prognostically unfavorable changes at diagnosis exhibited significantly increased rates of instability [56]. In fact, even normal karyotypes can become highly unstable and develop into complex karyotypes during disease progression [53]. An interesting and potentially clinically relevant question is whether and how often karyotypic evolution leads to a switch in cytogenetic risk group. While in one study this was the case for only 6/44 patients (14%; intermediate to unfavorable in all cases) [56], in another report, a transition from favorable to intermediate and from intermediate to unfavorable cytogenetics was found in 2 (12%) and 8 (47%) of 17 patients with karyotypic changes, respectively [55].

**Fig. 2:**
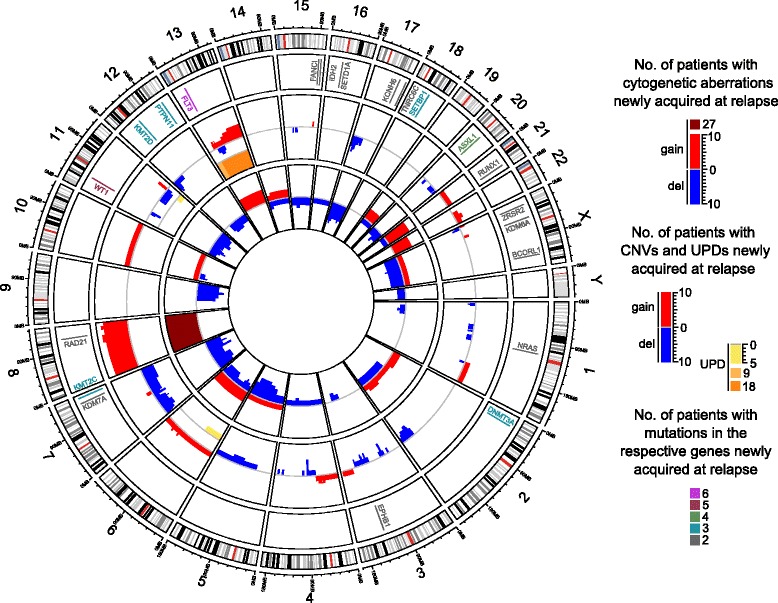
Circos plot summarizing genetic aberrations recurrently acquired at relapse in adult patients with non-APL AML. *Inner circle*, unbalanced cytogenetic aberrations newly acquired at relapse in at least 2 patients [53–56, 63, 68, 122]; *middle circle*, CNAs and UPDs newly acquired at relapse in at least 2 patients [64–69]. Within each type of aberration, overlapping lesions were considered recurrent events unless an aberration was reported only in 1 patient and became recurrent due to the same type of aberration affecting the corresponding entire chromosome in another single patient. For high patient numbers, different scales were used and patient numbers color-coded as indicated in the graphical legend. *Outer circle*, genes affected by SNVs or indels in a relapse-specific manner in at least 2 patients according to next generation sequencing-based studies [34, 36, 38, 39, 68, 95, 96]. The plot was constructed using the R package “circlize” [123]. Genomic positions of genes and chromosome bands were retrieved from the UCSC genome browser, human genome version hg19. Detailed data are provided in Additional file 1: Table S1A, Additional file 2: Table S2A, and Additional file 3: Table S3A. These also include studies containing exclusively pediatric patients or patients with APL, which were not considered for this figure. Recurrently gained aberrations are shown in this graph irrespective of whether or not they were recurrently lost in other patients; information about recurrent loss at relapse is provided in Additional file 1: Table S1B, Additional file 2: Table S2B, and Additional file 3: Table S3B

Aberrations newly acquired at relapse in a recurrent manner are summarized in Fig. 2 and Additional file 1: Table S1A, those repeatedly lost at relapse are listed in Additional file 1: Table S1B. As is evident from Additional file 1: Table S1A, each of the above cited studies found several recurrently gained aberrations, but only few of these were concordant between the different reports. Among the latter were trisomy 8, trisomy 21, and deletions affecting the long arm of chromosome 9. However, the trisomies were also lost at relapse in several cases (Additional file 1: Table S1B), and neither they nor del(9q) were unequivocally associated with a particularly poor response to therapy when present at diagnosis [18, 19, 57, 58], thus questioning their potential roles as drivers of therapy resistance and relapse. Deletions of chromosome bands 11p13 and 11q23 were also recurrently gained at relapse in more than one study. They were also reported in diagnostic samples [59–61], but to the best of authors’ knowledge, their prognostic significance is not known. Any conclusion about their potential contribution to therapy refractoriness and relapse therefore has to await further investigations. In contrast, deletions affecting the long arms of chromosomes 5 and 7 were not only recurrently acquired at relapse (Additional file 1: Table S1A, Fig. 2) but also associated with a poor outcome when present already at diagnosis [19, 62], making them potentially interesting candidates for lesions with a role in therapy resistance and disease recurrence.

Some studies also investigated possible associations of karyotypic changes between diagnosis and relapse, or of chromosome aberrations present at relapse, with various outcome parameters. Two independent studies, including 67 and 56 patients, respectively, reported that the duration of first remission (CR1), or the time from diagnosis to first relapse (TTR), did not differ significantly between patients with a normal karyotype at both diagnosis and relapse and patients who progressed from a normal to an abnormal karyotype [54, 56]. For patients with an abnormal karyotype at diagnosis, however, the length of CR1 was found to be independent of karyotypic stability in a cohort of 101 patients [54], while TTR was reported to be significantly shorter in cases with evolution of an abnormal karyotype or with an unrelated abnormal karyotype at relapse, compared to that in cases with regression or no alteration of an abnormal karyotype in a group of 61 patients [56]. Investigating the response to treatment for first relapse, Estey et al. found no difference regarding the likelihood to achieve CR2 or its duration between 47 patients who exhibited a normal karyotype at both diagnosis and relapse and 20 patients who progressed from a normal to an abnormal karyotype [54]. In contrast, Wang et al. reported that event-free survival (EFS) after relapse was significantly shorter in 30 patients with a normal karyotype at diagnosis and an abnormal karyotype at relapse than in 30 patients with a stable normal karyotype [63]. Similarly, among 45 patients with various karyotypes at diagnosis, the overall response to treatment for first relapse was significantly lower in the 17 cases with an unstable karyotype, and karyotypic stability was the only independent predictor of overall survival (OS) and EFS in multivariate analyses [55]. Finally, Kern et al., investigating a cohort of 120 patients, found that only karyotype at relapse, but not at diagnosis, significantly influenced response to treatment of relapsed disease. Furthermore, even though an unfavorable karyotype at diagnosis was associated with shorter OS and EFS as compared to intermediate or good risk karyotypes, the differences were even stronger when considering the karyotype at relapse [56]. Due to the heterogeneity of these studies regarding patient populations as well as influence and outcome parameters, a clear understanding of the roles of karyotypic stability and of karyotype at relapse with respect to the prognosis of AML will have to await additional studies.

## Changes in copy number alterations and uniparental isodisomies between diagnosis and relapse of AML

Several studies employed single nucleotide polymorphism (SNP) arrays to compare acquired CNAs (aCNAs; i.e., gains and deletions), and copy neutral losses of heterozygosity (i.e., UPDs) between samples collected from AML patients (*n* = 11–53) at presentation and recurrence. aCNAs/UPDs were rather infrequent in AML, with an average of <1–~5 such events per sample, but their number increased significantly from diagnosis to relapse [64–69]. In contrast, a whole exome sequencing (WES) study on 20 cytogenetically heterogeneous pediatric AML patients found that aCNAs/UPDs were gained and lost at relapse at similar rates, and only about 20% of these events persisted between presentation and recurrence [70]. Whether the discrepancies between the WES and the array-based studies are due to differences in methodologies and/or patient populations remains to be established.

Some aCNAs/UPDs affecting specific chromosomal regions were acquired at relapse in a recurrent manner (Fig. 2, Additional file 2: Table S2A), but, as with aberrations detected via cytogenetic analysis, only a limited number of these were identified as recurrent in more than one study. Among these are del(2q33.3), del(3p14.2), del(4q22.1), del(12p13), UPD(13q), and del(17p13) (Additional file 2: Table S2A). Deletions of chromosome bands 2q33, 3p14, and 4q22 have been reported infrequently at diagnosis of AML [59], and to the best of the authors’ knowledge, little if any information is available regarding their potential prognostic significance. Del(12p13) was frequently observed in diagnostic samples from patients with a complex karyotype, which is per se indicative of a poor prognosis, and candidate tumor suppressor genes have been mapped to the affected region [71]. Acquisition of UPD(13q) at relapse in most cases transformed a *FLT3* internal tandem duplication (ITD) that had existed in a heterozygous state at diagnosis to homozygosity [64]; the presence of comparable lesions already at diagnosis was associated with poor responsiveness to therapy [72, 73]. Finally, deletion of the tumor suppressor gene *TP53* in chromosome band 17p13 was frequent in cytogenetically complex diagnostic samples and independently predicted poor survival on the background of both complex and non-complex karyotypes [19, 74]. Acquisition of a comparable lesion, namely, monosomy 17, at relapse was also repeatedly observed by cytogenetic analysis (Additional file 1: Table S1A). UPD(13q), del(17p13), and possibly some of the other abovementioned aberrations can therefore be considered interesting candidates for a role in therapy resistance and relapse.

## Changes in the mutational status of known leukemia driver genes between diagnosis and relapse of AML

SNVs or indels affecting genes that are recurrently mutated in diagnostic AML samples are considered drivers of the leukemogenic process, may be predictive of outcome, and, if stable during the course of disease, may serve as markers for disease monitoring [18, 19, 75]. Furthermore, such mutations, if newly acquired at relapse, might contribute to therapy resistance associated with this stage, especially if their presence at diagnosis is also associated with poor outcome, as is the case, e.g., for the *FLT3* internal tandem duplication (*FLT3*-ITD) and for mutations in *ASXL1*, *DNMT3A*, and *RUNX1* [18, 19]. For these reasons, a number of studies have investigated the presence or absence of such mutations at different stages of AML. As summarized in Table 1, *FLT3*-ITD and *FLT3* tyrosine kinase domain (*FLT3*-TKD) mutations, as well as *RAS*, *TP53*, *WT1*, and *IDH1* mutations were recurrently gained at relapse of AML [65, 69, 76–84]. Furthermore, the *FLT3*-ITD/wild-type ratio, which represents an additional prognostic factor, was increased at relapse in several patients [81, 85]. However, all of these mutations, except for those in *TP53*, were also recurrently lost at relapse (Table 1, Additional file 3: Table S3B) [65, 69, 76, 77, 79–84, 86, 87], which makes a strong contribution to therapy refractoriness at disease recurrence less plausible.

SNVs/indels can be measured with higher sensitivity than molecularly poorly characterized cytogenetic aberrations or CNVs/UPDs. Some authors therefore asked whether their new appearance at relapse was due to the expansion of a cell population present at diagnosis but too small for detection with standard methods, or to actual de novo mutation. While a radioactive PCR assay detecting the *FLT3*-ITD with a sensitivity of 1/200 supported the latter possibility in 3/3 investigated patients [80], patient-specific *FLT3*-ITD qRT-PCR assays with a sensitivity of 10^−4^–10^−5^ provided evidence for the former scenario in 4/6 tested patients [47]. Similarly, mutations present at relapse and undetectable in the leukemic bulk at diagnosis could be traced back to flow sorted subpopulations of 5/7 presentation samples [46]. Targeted resequencing (median coverage, 20.000) in 3 patients who relapsed within 1 year revealed that some of the putative relapse-specific mutations were present at low ratios already at diagnosis, while others remained undetectable even at this level of sensitivity [36]. In contrast, in 5 patients relapsing after more than 5 years, none of the relapse-specific mutations was detected at diagnosis using targeted resequencing at a sensitivity of 0.001 [38].

*FLT3*-ITD alleles have varying lengths and insertion sites, facilitating detailed molecular analyses that revealed complex and highly dynamic clonal patterns. Patients displayed up to three different alleles at a given time point during the course of their disease. In some cases, only one out of two or three mutations present at diagnosis was preserved at relapse and could be derived from either the major or a minor clone present at diagnosis. Some patients lost one of their diagnostic alleles at relapse and concomitantly acquired a new one. Others had only wild-type alleles at diagnosis but relapsed with two different ITD alleles, or had one mutation type at diagnosis and relapsed with the same allele in addition to a newly gained one [81, 85, 88]. Similarly, complex patterns of losses and gains of mutations were reported for the *RUNX1* gene [89]. Single cell analysis further underscored the substantial clonal diversity in AML: in samples with two different *FLT3*-ITD alleles, single cells either had wild-type alleles only, or harbored one of the two mutant alleles in a homozygous or a heterozygous state but, interestingly, no single cell was compound heterozygous for the two ITD alleles. In contrast, in samples containing both *FLT3* and *NPM1* mutations, these occurred in all possible combinations [90].

Some authors also related mutational instability, or mutation status at relapse, to outcome parameters. In a study on 23 pediatric AML patients of all cytogenetic risk categories, cases with a mutational shift in *FLT3*, *RAS*, *PTPN11*, and/or *WT1* had significantly worse OS than those with mutational stability [84]. *FLT3*-ITD and *TP53* mutations at disease recurrence were significantly associated with short survival after relapse among 28 adult patients with cytogenetically heterogeneous AML [77]. Perhaps even more remarkably, in a cohort of 80 pediatric and adult patients with various karyotypes, *FLT3*-ITD status at relapse was associated with TTR more significantly than the same molecular feature at diagnosis [79], and among 69 patients with pediatric AML and mixed cytogenetics, *FLT3*-ITD and *WT1* mutations at relapse, but not at presentation, were significantly associated with shorter OS, with *FLT3*-ITD status at relapse confirmed as an independent prognostic parameter in multivariate analyses [83]. Even though it has to be kept in mind that the inclusion only of relapsing patients led to a skewing of the patient population at diagnosis in these studies, their results suggest that mutations existing at presentation in subpopulations too small for detection with standard methods, or even acquired only during therapy, may have a more important impact on outcome than the genotype of the bulk leukemic population at diagnosis. If this notion can be confirmed in larger patient cohorts, it may have important implications for the routine assessment of prognostically relevant mutations at diagnosis.

## Next generation sequencing to investigate SNVs and indels during the evolution of AML

In a seminal study published in 2012, Ding et al. established several important concepts regarding the evolution of AML from presentation to recurrence [34]. Matched constitutional (skin), diagnostic, and relapse samples from 8 adult patients with AML (7 with a normal karyotype, 1 with t(15;17)) were subjected to whole genome sequencing (WGS), followed by validation of variants through deep sequencing of captured targets. An average of 539 somatic mutations and structural variants in the non-repetitive regions of the genome, of which 21 affected protein coding regions, were identified per case. The majority of the mutations were shared between diagnosis and relapse, and only relatively small proportions were gained or lost at the latter stage [34]. All patients harbored between one and four mutation clusters at diagnosis, and all acquired additional mutations at relapse, although remarkably in three cases, none of these mutations were non-synonymous. Two major patterns of clonal evolution were observed: in 3 patients, the dominant clone at diagnosis gained additional mutations and evolved into the relapse clone, while in 5 patients, one or two minor subclone(s) carrying most, but not all, of the mutations present at diagnosis acquired new sequence variants and expanded at relapse [34]. Among the relapse-specific mutations, the proportion of transversions was significantly increased, suggesting that chemotherapy affected the mutation pattern and, through its mutagenic effects, may have directly contributed to therapy resistance [34].

Subsequent reports employing whole exome sequencing (WES) (usually followed by validation through independent methods) and/or targeted resequencing confirmed and extended these findings. Two WES studies, restricted to adult patients with *FLT3*-ITD-positive AML (*n* = 13) and core-binding factor AML (*n* = 10), respectively, found numbers of exonic mutations comparable to those in the earlier investigation. Again, the majority of these mutations persisted during disease progression, while some were specific to either diagnosis or relapse [39, 68]. These investigations also corroborated the increase in the proportion of transversions among relapse-specific mutations, as well as the previously described patterns of clonal evolution [39, 68]. As an extension to the latter aspect, some studies suggested that relapse clones may also evolve from preleukemic HSCs, thus uncovering another potential cellular origin of disease recurrence [36, 38, 69]. Along similar lines, the relatedness between leukemic clones at diagnosis and relapse decreased with increasing TTR: targeted resequencing of 122 recurrently mutated genes in paired samples from 22 patients with cytogenetically heterogeneous AML revealed a significantly larger number of retained mutations in patients relapsing after <3 years than in those relapsing after >5 years, while the number of gained or lost lesions behaved in the opposite manner. Nevertheless, no relation between either TTR or the extent of clonal evolution and response to therapy for recurrent disease was observed in this study [38]. This may be due to the small size of the patient cohort, however, because the duration of CR1 is a well-established prognostic parameter in relapsed AML [2].

Three studies applied WES to pediatric AML, two including each 4 [91, 92], and the third 20 [70], patients with variable karyotypes. Their findings essentially paralleled those in adult AML. As an interesting extension, Farrar et al. reported a median of 3.5 and 8 non-synonymous mutations in patients <2 and 2–17 years old, respectively, supporting the assumption that the majority of mutations present in leukemic cells are a result of aging, rather than causal contributors to tumorigenesis [70].

Due to its different biology and treatment modalities [8, 9, 93], mutational patterns in APL might be expected to differ from those of other AML cases. Indeed, in 222 partially paired samples from 200 patients with pediatric and adult APL, targeted resequencing showed that both at diagnosis and at relapse, the frequencies of some of the known AML driver mutations were distinct from those in the 180 diagnostic non-APL AML samples in the The Cancer Genome Atlas cohort [17, 94]. At recurrence, mutations in *PML* and *RARA* were repeatedly observed in patients treated with arsenic trioxide and *all-trans* retinoic acid, respectively [94]. WES on paired samples from 8 patients with a median age of 22.5 years revealed an average of 9.6 non-synonymous mutations per patient. As in non-APL AML, mutational load did not change significantly from diagnosis to relapse. Remarkably, in 2 of 8 patients, no SNVs/indels but only the *PML-RARA* fusion persisted between presentation and recurrence [94], suggesting that it was an early event in these cases.

In an approach somewhat different from the above discussed studies, Kim et al. used WES to track the course of disease of a patient presenting with cytogenetically normal AML at the age of 36 over 9 years, during which he experienced four CRs and four relapses [95]. Two findings of this study appear particularly noteworthy: Firstly, a single cellular clone constituted the leukemic population from relapses two through four, raising the question which (molecular) events caused therapy resistance at the final relapse (the unconvincing candidates captured by WES were loss of mutations in *OR2T12* and *NPM1*, gain of a synonymous mutation in *VTN*, and a moderate increase in the VAF of a splicing mutation in *TMEM63C*). Secondly, a clone containing the *DNMT3A*-R882H mutation of the founding clone, along with five additional variants, expanded strongly during CRs two to four to reach VAFs of up to 50% (indicating monoclonality) and decreased, but was not eliminated, during subsequent relapses [95]. Similar observations were reported for 5 of 15 adult de novo AML patients investigated by WES: clones defined by somatic variants not present in the AML clones yet detectable at VAFs <1% at diagnosis, expanded 30–150-fold within 1–2 months after initiation of therapy and persisted at similar or increasing levels through observation periods of 161–544 days [96]. The clinical implications of this phenomenon are presently unclear.

A unique subgroup among patients with AML are those with familial disease due to predisposing germ line mutations, e.g., in *CEBPA*. In a cohort comprising ten pedigrees with this condition, patients presented with AML at a median age of 24.5 years [97]. WES on nine leukemic samples identified on average 17.8 tumor-specific mutations with predicted translational consequences per patient [97], comparable to the numbers in sporadic AML. The somatic mutations affecting the second *CEBPA* allele, as well as additional SNVs identified by WES, were discordant between diagnosis and recurrence, suggesting that in this condition, recurrences more commonly represent new leukemic episodes rather than relapses of the original disease, in agreement with the clinical observation that they frequently retain therapy sensitivity [97].

In summary, next generation sequencing-based methods have yielded important insights into the biology and evolution of AML. However, other than in T cell acute lymphoblastic leukemia (T-ALL), where activating mutations in the gene encoding the drug inactivating 5′-nucleotidase NT5C2 were acquired in almost one fifth of patients at disease recurrence [51, 52], no striking candidate genes, mutations in which would be recurrently gained and rarely or never lost at relapse of AML and plausibly contribute to therapy resistance, were so far identified (Additional file 3: Table S3A, B). As possible exceptions, mutations in *ASXL1*, *SETBP1*, and *ZRSR2* were recurrently gained but not recurrently lost at relapse (Table 1, Additional file 3: Table S3A, B) and were associated with a poor outcome when present at diagnosis [18, 19, 98]. However, the numbers of patients who acquired mutations in these genes were small (two to four cases; Additional file 3: Table S3A).

Even though it remains possible that application of unbiased methods like WES or WGS to larger, more homogeneous patient cohorts will lead to the identification of (additional) candidate driver mutations of relapse, a universal role of SNVs or indels—at least in their “classical” mode of action—in the evolution of therapy resistance is also questioned by observations that disease can recur in a refractory manner without the acquisition of additional non-synonymous mutations [34, 92, 95]. Therefore, mutations with consequences other than a change in primary protein structure, e.g., regulatory variants, and/or molecular events not captured by genome sequencing methodologies, e.g., epigenetic or gene expression changes, may play important or even dominant roles in the development of therapy resistance and relapse.

## Changes in the methylation of gene regulatory regions between diagnosis and relapse of AML

Alterations in DNA methylation are common in myeloid malignancies, and demethylating agents play a role in their clinical management [99]. To explore the relevance of changes in DNA methylation at relapse of AML, Li et al. performed genome-wide methylome analysis through Enhanced Reduced Representation Bisulfite Sequencing (ERRBS) on paired diagnosis and relapse samples from 138 cytogenetically heterogeneous patients; 48 of these were additionally subjected to WES [100]. Eloci were defined as sequences of four consecutive CpGs exhibiting a significant shift of the entropy of their methylation status as compared to normal bone marrow. While eloci were significantly overrepresented at CpG islands and promoters at diagnosis, they were enriched in intronic and intergenic regions at relapse. Neither overall mutation burden nor the presence of mutations in specific genes (e.g., epigenetic modifier genes) was significantly associated with levels of eloci per million loci. However, in contrast to somatic mutation load, high eloci per million loci values at diagnosis were significantly associated with shorter TTR, a relation that was most significant for promoter-associated eloci [100].

Kröger et al. focussed on nine genes whose CpG islands had previously been shown to be hypermethylated in primary and cultured malignant hematopoietic cells, and probed them by bisulfite pyrosequencing in paired diagnostic and relapse samples from 30 patients with cytogenetically heterogeneous AML [101]. The median number of methylated genes increased from four at presentation to six at recurrence, and a significant increase in methylation density at relapse was observed for the CpG islands of the *CDH13*, *NPM2*, *PGRA*, *HIN1*, *SLC26A4*, and *CDKN2B* genes [101]. Methylation of the *CDKN2B* CpG island also increased significantly from 48/77 (62%) cases at presentation to 30/36 (83%) at relapse of APL [102].

## Changes in the expression of protein coding and microRNA genes between diagnosis and relapse of AML

Altered expression of certain genes contributes to leukemogenesis, and the mRNAs levels of single genes as well as the presence of specific multi-gene expression signatures at diagnosis are predictive of outcome in AML [20–22, 103–105], raising the possibility that recurrent gene expression changes between diagnosis and relapse may also contribute to therapy resistance at the latter stage. Some studies suggested that single genes, selected for investigation based on prescreening data from independent experimental systems, e.g., *TGM2*, *CDK1*, miR-331-5p, and miR-27a, were significantly deregulated between diagnosis and relapse of AML [106–108]. Others pursued large-scale approaches to identify genes differentially expressed between the two disease stages [84, 109–111]. Even though these investigations, performed on limited numbers of only partially matched and mostly cytogenetically heterogeneous samples, identified genes differentially expressed between diagnosis and relapse, correction for multiple hypothesis testing was either not performed or not passed by any of the probed genes. In contrast, Hackl et al., restricting their analyses to cytogenetically normal AML and using 11 paired diagnostic and relapse samples for genome-wide gene expression profiling, discovered 536 and 551 genes that were up- and downregulated at relapse, respectively, at a false discovery rate (FDR) of <10% [112]. Supporting the notion that specific gene expression patterns may contribute to therapy resistance in relapsed AML, previously defined diagnostic expression signatures associated with poor outcome in this disease [21, 22, 113] and/or with LSCs [13] were significantly enriched in the relapse-associated gene expression profile [112]. Consistent with these findings, Ho et al. recently reported a 9- to 90-fold increase in the frequency of functional LSCs, measured by limiting dilution transplantation into NSG mice, in relapsed versus diagnostic samples from 5 patients with AML [114].

In addition to protein-coding genes, microRNA genes may be differentially expressed between diagnosis and relapse of AML: a Taqman low-density array screen on six paired samples from pediatric patients with *MLL* rearrangements uncovered 53 microRNAs that were up-, but none that were down-, regulated at relapse at an FDR of <10%, among them the miR-106b~25 cluster. The expression pattern of about half of the tested microRNAs could be confirmed by qRT-PCR in the original, plus eight additional, sample pairs [115].

While all of the abovementioned studies measured gene expression in a static manner, expression changes elicited by chemotherapeutic drugs may also play a role in the response to them. Therefore, Fisser et al. searched for genes that were induced by cytostatics in the human myeloid cell line U937. *CADM1* was upregulated upon exposure to cytosine arabinoside, daunorubicin, or etoposide, and its experimental expression in U937 cells led to an increase in the proportion of apoptotic cells [116]. Corroborating its suspected role in chemotherapy induced cell death, *CADM1* was upregulated in response to in vitro exposure to cytosine arabinoside in 3/3 primary AML samples from the time of diagnosis, but in none of the matched relapse samples [116].

## Miscellaneous molecular parameters that changed between diagnosis and relapse of AML

The following chapter briefly summarizes several studies that compared molecular parameters not readily attributable to any of the above categories between diagnosis and relapse of AML. In the first of these, Shlush et al. inferred the number of cell divisions that had occurred in a cell population from single cell microsatellite data and concluded that in two investigated patients with AML, proliferative activity was higher at diagnosis than at relapse [117]. Another report, based on analyses of the mutation and promoter methylation status of the *MSH2* and *MLH1* genes in unpaired diagnostic, relapsed, and refractory samples from 53 pediatric and adult patients with AML, suggested that aberrations affecting mismatch repair genes were significantly more frequent in refractory/relapsed samples than in diagnostic ones (12/25 versus 6/28) [118]. Zhou et al. measured various parameters related to oxidative stress in paired presentation and recurrence samples obtained from 102 adult patients with AML, as well as in an equal number of age-matched healthy controls, and concluded that relapse was associated with an increase in oxidative stress [119]. Finally, mitochondrial priming, measured via molecular indicators of programmed cell death elicited by proapoptotic BH3-only peptides and proposed to reflect cellular propensity to undergo apoptosis in response to appropriate stimuli, was significantly decreased in relapse samples from six patients as compared to the matched diagnostic specimens [120].

## Conclusions

**Fig. 3:**
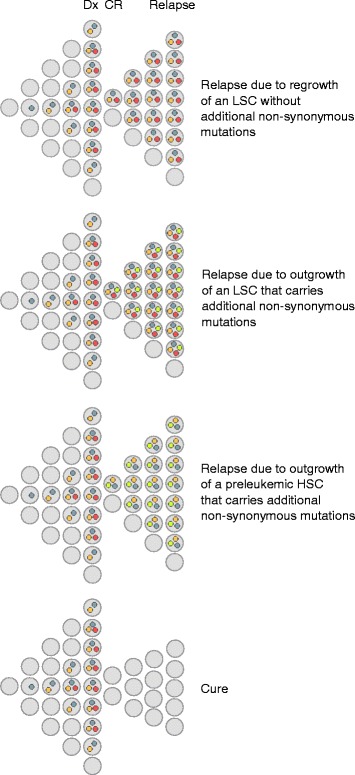
Different pathways leading to relapse of AML. *Gray dots*, age-related, pathogenetically irrelevant passenger mutations; *orange dots*, early (pre-) leukemic driver mutations; *red dots*, late leukemic driver mutations; *bright yellow dots*, non-synonymous mutations newly acquired at relapse. All HSCs are assumed to accumulate mostly innocuous mutations during aging; only mutations that would be found as passenger mutations in AML are depicted in the figure. The figure does not intend to illustrate the duration of CR, or the presence or absence of minimal residual disease detectable by routine methods. *Dx* diagnosis, *CR* complete remission, *LSC* leukemic stem cell, *HSC* hematopoietic stem cell

Based on mutational profiles and clonal dynamics observed in patients with AML, three major pathways to relapse have been proposed [18, 36, 38, 44, 69] (Fig. 3). Each of these appears to be taken in a subset of cases. In patients whose disease follows the first trajectory, few if any genetic alterations with presumed functional relevance distinguish relapse from diagnostic samples; relapse is supposed to be due to the regrowth of an LSC present already at diagnosis and often follows a short remission [44]. In patients who nevertheless experience a longer remission [38, 92, 95], genetic changes not captured by the methods applied, changes with less obvious functional consequences (e.g., mutations in regulatory regions), and/or epigenetic or gene expression alterations may contribute to increased therapy resistance at relapse. The second pathway is reflected by a scenario in which a diagnostic clone harboring both early (preleukemic) and late leukemogenic driver mutations reappears with additional, newly gained mutations at relapse; disease recurrence is therefore due to the evolution of an LSC. In the third pathway, the diagnostic clone(s) contain(s) both early and late driver mutations, but only the early, preleukemic lesions, along with newly acquired mutations, are present at relapse, consistent with relapse originating from a preleukemic HSC rather than an LSC.

In some patients with apparent relapse-specific mutations, these could retrospectively be demonstrated to have been present at very low levels also in the diagnostic sample, while in others, they were undetectable at this time point even with highly sensitive methods [36, 38, 46, 47], suggesting that they either emerged only during therapy, or that the stem cells harboring them were proliferatively inactive at diagnosis. Irrespectively, by analogy to the role of genetic and molecular alterations recurrently present at diagnosis of AML as drivers of leukemogenesis, lesions recurrently acquired at relapse can be expected to contribute to the increased aggressiveness and therapy resistance associated with this disease stage. In addition, similar to their counterparts at diagnosis [8, 9, 28, 29], drivers of relapse represent potential targets for therapeutics for the treatment of recurring disease, or, ideally, for the preemptive eradication of initially small therapy-resistant cell populations so as to prevent the occurrence of relapse altogether.

Indeed, the studies summarized above have identified some aberrations that were newly gained at relapse in a recurrent manner and may plausibly contribute to therapy resistance at this stage, like deletions of the *TP53* gene in chromosome band 17p13 or point mutations in *ASXL1*. However, these lesions were present only in a low single-digit percentage of the investigated patients. In contrast, in patients selected for the presence of specific genetic features at diagnosis, the expression of certain mRNAs or miRNAs changed between presentation and recurrence in a statistically significant manner, implying that these alterations occurred in a large proportion of cases. Furthermore, the mRNA expression profile associated with relapse of normal karyotype AML was enriched for gene signatures associated with LSCs and with a poor prognosis, suggesting that some of its member genes may contribute to therapy resistance at relapse. However, it remains to be shown whether these gene expression changes are sufficiently uniform among single leukemic cells and sufficiently stable to represent useful therapeutic targets.

In summary, even though so far only a limited number of studies has addressed the molecular alterations specifically acquired at relapse of AML, important progress has been made in understanding the genetics and molecular biology associated with this largely therapy-resistant disease stage. Despite of these advances, many open questions remain. Among these are the extent to which genetic alterations present at diagnosis and variations in treatment determine which additional lesions are able to allow leukemic cells to survive therapy and regrow at relapse. The possible functional role of mutations without predicted translational consequences warrants further exploration [121]. The genetic, epigenetic, and gene expression alterations between diagnosis and relapse of AML need to be investigated in larger patient cohorts, which may need to be more homogeneous in terms of, e.g., antecedent clonal hematopoiesis of indeterminate potential, genetics at diagnosis, and/or treatment. In an ideal scenario, all types of molecular and genetic alterations would be probed in the same set of paired samples, and the resulting data integrated to potentially identify different sorts of lesions affecting the same genes or pathways. Functional analysis of these candidate genes and/or pathways with respect to their involvement in therapy resistance is expected to identify targets for novel therapeutics able to substantially improve outcome in patients with AML.

## Additional files

Additional file 1: Table S1. Cytogenetic aberrations recurrently acquired (Table S1A) or lost (Table S1B) at relapse of AML. (XLSX 25 kb)
Additional file 2: Table S2. CNAs and UPDs recurrently acquired (Table S2A) or lost (Table S2B) at relapse of AML. (XLSX 25 kb)
Additional file 3: Table S3. Non-synonymous mutations recurrently acquired (Table S3A) or lost (Table S3B) at relapse of AML as determined by next generation sequencing based methods. (XLSX 22 kb)

## Abbreviations

aCNA: Acquired copy number alteration
AML: Acute myeloid leukemia
APL: Acute promyelocytic leukemia
CNA: Copy number alteration
CR: Complete remission
EFS: Event-free survival
FDR: False discovery rate
HSC: Hematopoietic stem cell
HSPC: Hematopoietic stem and progenitor cell
indel: Small insertion or deletion
ITD: Internal tandem duplication
LSC: Leukemic stem cell
OS: Overall survival
RARA: Retinoic acid receptor alpha
SNP: Single nucleotide polymorphism
SNV: Single nucleotide variant
T-ALL: T cell acute lymphoblastic leukemia
TKD: Tyrosine kinase domain
TTR: Time to relapse
UPD: Uniparental isodisomy
VAF: Variant allele frequency
WES: Whole exome sequencing
WGS: Whole genome sequencing
